# Evaluating the Impact of Telemedicine on Diabetes Management in Rural Communities: A Systematic Review

**DOI:** 10.7759/cureus.64928

**Published:** 2024-07-19

**Authors:** Osamah AlQassab, Tatchaya Kanthajan, Manorama Pandey, Aida J Francis, Chithra Sreenivasan, Aneri Parikh, Marcellina Nwosu

**Affiliations:** 1 Internal Medicine, California Institute of Behavioral Neurosciences & Psychology, Fairfield, USA

**Keywords:** healthcare disparity, internal medicine (general medicine), rural health services, rural area, glycated hemoglobin (hba1c), diabetes self-management, glycemic control, diabetes mellitus management, telehealth, telemedicine

## Abstract

Telemedicine is the delivery of healthcare services using information and communication technologies to diagnose, treat, and prevent diseases. The COVID-19 pandemic has accelerated the adoption of telemedicine, transforming how healthcare is delivered, especially in remote and underserved areas. Despite its potential, no systematic reviews have been conducted in the last five years to assess the effectiveness of telemedicine for managing diabetes in rural populations. This review addresses this gap by evaluating studies on telemedicine's impact on glycemic control among diabetic patients in these settings. We searched five databases: PubMed, Google Scholar, ClinicalTrials.gov, ScienceDirect, and Science.gov, covering studies published in the last five years. Of the 331 articles identified, 10 met our inclusion criteria: English-language studies from the past five years involving adults in rural areas or comparing rural and urban settings, focusing on telemedicine's impact on glycemic control in diabetic patients. In many studies, the findings revealed that telemedicine interventions integrated into structured programs significantly improved HbA1c levels. Successful implementation requires local infrastructure and consistent patient-provider interactions, although increased healthcare provider workloads may affect sustainability. Telemedicine alone was less effective for patients with complex comorbidities, suggesting that a combined approach with in-person visits may be more effective. This review highlights telemedicine's potential to replace routine in-person visits for diabetes management in rural areas, demonstrating significant improvements in HbA1c levels, medication adherence, and timely care management support. Future research should focus on randomized controlled trials in rural settings, hybrid care models that optimize in-person visit frequency and remote monitoring, and addressing technological challenges such as broadband access and platform usability to ensure sustainable telehealth interventions.

## Introduction and background

Diabetes mellitus impacts approximately 537 million people worldwide, making it a global health concern, and experts expect this number to rise to 783 million by 2045. Studies showed a rise in the prevalence of diabetes in rural areas, and nowadays, out of every three individuals affected by diabetes, one lives in a rural setting (176 million) [1]. Rural populations face many challenges that can prevent better glycemic control, such as geographic isolation, health worker shortages, and lower health spending compared to urban populations [2]. The prevalence of diabetes is estimated to be 38.4 million in the United States and is one of the top chronic conditions that require proper management and investigation [3]. In the United States, studies showed that there is a 17% higher prevalence of diabetes in rural areas compared to urban areas, with poorer outcomes. The elevated prevalence of diabetes positions it as the third most critical rural health priority in 2020. While diabetes mortality rates have declined in urban areas over the past two decades, rural areas have shown the opposite [4].

The World Health Organization (WHO) defines telemedicine as the delivery of healthcare services, where distance is a critical factor, by all healthcare professionals (HCPs) using information and communication technologies for the exchange of valid information for the diagnosis, treatment, and prevention of disease and injuries, research and evaluation, and for the continuing education of HCPs, all in the interests of progressing the health of individuals and their communities [5]. It also includes remote monitoring, data exchange, and service provided by the clinician to the patient [6]. After the COVID-19 pandemic, telemedicine to reach patients increased significantly and successfully led to positive outcomes in controlling various disease conditions [7]. A meta-analysis of four clinical studies demonstrated a positive effect of telemedicine on glycemic control self-management in the short term within a primary healthcare setting [8]. Another meta-analysis involving 107 studies (20,501 participants) demonstrated that telemedicine successfully lowered HbA1c levels significantly compared to standard care, with a mean difference ranging from 0.37% to 0.71%. Among the different types of telemedicine, teleconsultation was the most effective strategy [9]. Finally, telemedicine for diabetes care has effectively lowered HbA1c levels in several randomized controlled trials [10-12].

Despite the promising outcomes associated with telemedicine, several significant gaps in rural areas still need to be addressed: limited internet access, lower technological services, and socioeconomic barriers can decrease the utilization of telemedicine services in these areas [13]. While telemedicine has shown efficacy in reducing HbA1c levels, as evidenced by several meta-analyses and clinical trials [9-12], the variability in study designs and patient populations makes it challenging to generalize these findings across all rural areas [14]. There is also a need for more real-world implementation studies to understand the practical challenges and facilitators of telemedicine in these communities [15]. The integration of telemedicine with existing healthcare systems and the continuity of care post-intervention remain areas requiring further exploration. Many studies highlight the initial success of telemedicine programs but need long-term follow-up data to ascertain the sustainability of these interventions [16].

This systematic review aims to illuminate existing barriers by analyzing the current evidence to evaluate the implementation and effectiveness of telemedicine in rural diabetes management. Notably, our research is the first in the past five years to examine the efficacy of telemedicine in improving glycemic control among diabetic patients in rural areas.

## Review

Methodology

This systematic review was conducted using the Preferred Reporting Items for Systematic Review and Meta-Analyses (PRISMA) 2020 guidelines [17].

Selection and data collection process

The search strategy involved a careful and systematic approach to identify relevant articles for this systematic review. Our research question focused on evaluating the effectiveness of telemedicine in improving HbA1c levels among diabetic patients in rural areas. We had three main concepts: digital health interventions, diabetes mellitus, and rural areas. The selection process involved multiple reviewers screening titles and abstracts. Our initial search yielded 331 articles, which were copied to EndNote (Clarivate, Philadelphia, Pennsylvania) and then transferred to an MS Excel sheet (Microsoft Corporation, Redmond, Washington) for duplicate removal. This resulted in 306 articles, which underwent thorough assessment, including reading abstracts and full texts when necessary. Articles whose titles and abstracts did not address our research question were excluded. The final 65 articles from the screening process underwent a detailed review of their entire texts to assess their eligibility.

Inclusion and exclusion criteria

In this systematic review, we included studies based on the following criteria: articles written in English, published in the last five years, and involving individuals aged 18 or older. The studies had to be conducted in rural areas or include comparisons between rural and urban areas. Specifically, we looked for studies that addressed the impact of digital health interventions on glycemic control in diabetic patients residing in rural areas.

Search sources and strategy

A comprehensive search was conducted to identify relevant studies for this systematic review. We employed several search techniques, including the building block method, the Boolean search strategy, and the snowball sampling technique. Five databases (PubMed, Google Scholar, ClinicalTrials.gov, ScienceDirect, and Science.gov) were searched using telemedicine, rural populations, and glycemic control keywords. Specific terms included "telemedicine," "mHealth," "digital health," "remote monitoring," "health apps," "glycemic control," "HbA1c," "diabetes complications," and "rural health." The final search was completed on April 26, 2024. All the search strategies, the databases used, and the identified number of papers for each database are shown in Table 1.

**Table 1:** Keywords used for search strategies and the number of papers identified MeSH: medical subject heading; PMC: PubMed Central

Databases	Keywords	Number of Articles
PubMed (MeSH)	"("telemedicine" OR "mHealth" OR "digital health" OR "remote monitoring" OR "health apps")" AND "("glycemic control" OR "HbA1c" OR "diabetes complications" OR "blood sugar management")" AND "("rural" OR "rural health" OR "rural population" OR "remote areas")"	32 articles
Google Scholar	"("telemedicine" OR "mHealth" OR "digital health" OR "remote monitoring" OR "health apps")" AND "("glycemic control" OR "HbA1c" OR "diabetes complications" OR "blood sugar management")" AND "("rural" OR "rural health" OR "rural population" OR "remote areas")"	15 articles
Clinical Trials.gov	Condition: diabetes mellitus Other terms: ("glycemic control" OR "HbA1c" OR "diabetes complications" OR "blood sugar management") Intervention/treatment: ("telemedicine" OR "mHealth" OR "digital health" OR "remote monitoring" OR "health apps")	32 articles
ScienceDirect	Telemedicine and Diabetes Mellitus and Glycemic Control and Rural Population	93 articles
Science.gov	Telemedicine and Diabetes Mellitus and Glycemic Control and Rural Population	159 articles

Quality appraisal

We used the Newcastle-Ottawa scale for the quality assessment of observational, non-randomized trials, and cohort studies, the Cochrane Risk of Bias tool for randomized controlled trials, and the Mixed-Methods Appraisal Tool (MMAT) for one mixed-methods study. Two researchers assessed the final 10 articles to ensure greater credibility in the final evaluation.

Results

**Figure 1:**
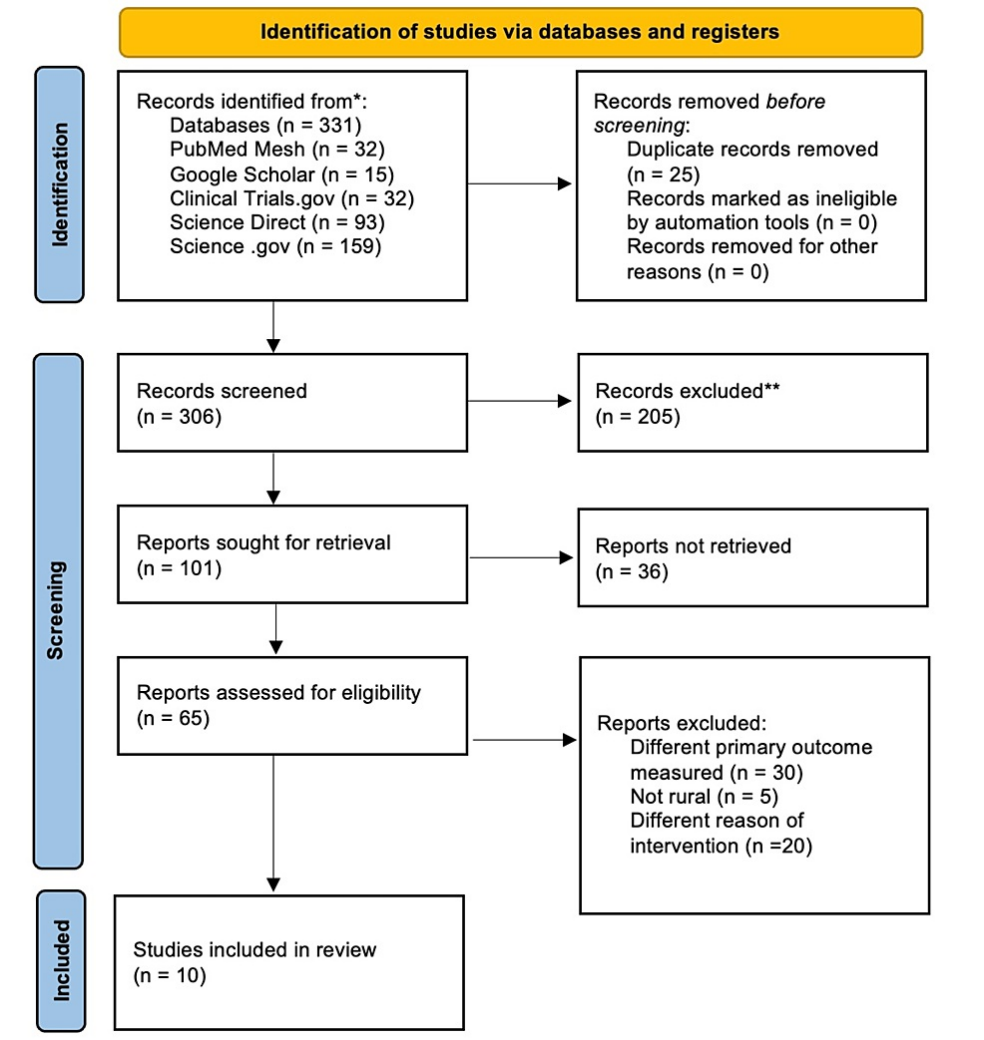
PRISMA 2020 flowchart depicting the process for the article selection *Consider, if feasible to do so, reporting the number of records identified from each database or register searched (rather than the total number across all databases/registers) **If automation tools were used, indicate how many records were excluded by a human and how many were excluded by automation tools PRISMA: Preferred Reporting Items for Systematic Review and Meta-Analysis; MeSH: medical subject heading; PMC: PubMed Central

A total of 331 articles were initially identified in our primary search. From these, 25 duplicate articles were removed, leaving 306 articles. These 306 articles underwent a thorough screening process, during which their titles and abstracts were read as needed. This process resulted in the removal of 205 articles. The remaining 101 articles were then subjected to a review of their abstracts, leading to 65 articles being selected for full-text review. Ultimately, we finalized 10 articles for inclusion in this study. The study selection process is depicted in the PRISMA flowchart (Figure 1).

Study characteristics

This systematic review includes one randomized clinical trial [18], five retrospective cohort studies [19-23], three prospective cohort studies [13,24,25], and one mixed-methods implementation study [26]. These articles' interventions consisted mainly of remote monitoring, self-management education, and telemedicine consultation. The participants were mainly affected by type 2 diabetes mellitus (T2DM), with a mean age of 58.7 ± 9.5 years. Most of the articles focused on rural, geographically isolated areas, with some comparing rural and urban areas. Finally, the demographics of the participants range differently based on the study design; for example, studies that focused on veterans contained more males than females, whereas other studies contained an equal portion. A summary of the included studies, assessing their interventions, study types, and measured outcomes, is presented in Table 2.

**Table 2:** Summary of the included studies V-IMPACT: virtual integrated multisite patient aligned care teams; DCN: diabetes care network; DC: diabetes consultation; DSME: diabetes self-management education; SMBG: structured self-monitoring of blood glucose; BGM: blood glucose meter; ACDC: advanced comprehensive diabetes care

Author and Year	Intervention	Sample Size	Gender	Mean Age	Rurality	Study Design	Results	Conclusion
Baum et al., 2023 [19]	Pharmacist-provided telehealth diabetes management for veterans	522	-	-	76.6%	Retrospective chart review	72% maintained HbA1c control, 12.2% improved HbA1c, 15.7% worsened HbA1c.	Glycemic control was maintained or improved in 84.2% of patients, demonstrating the effectiveness of telehealth in reaching rural veterans.
Davis et al., 2019 [24]	Telehealth for chronic care management in a rural setting	115	Males: 31% Females: 69%	53.6 ± 11.9	100%	Prospective, longitudinal cohort study	Mean HbA1c reduced significantly from 9.5% to 7.9% (p < 0.001).	Telehealth and remote patient monitoring are effective tools for improving diabetes management and outcomes in rural populations, demonstrating feasibility and benefits in underserved areas.
Lu et al., 2021 [20]	The V-IMPACT program utilized videoconferencing to connect rural veterans with primary care providers	9010	Males: 96% Females: 4.4%	68.4 ± 10.3	75%	Retrospective cohort study	HbA1c reduction: V-IMPACT group (-0.055%, 95% CI: -0.065 to -0.045) vs. Usual care (-0.047%, 95% CI: -0.057 to -0.037). Significant increases in statin and ACE/ARB prescriptions, and yearly microalbuminuria testing in the intervention group (p < 0.01).	There were no differences in HbA1c. The quality of diabetes care delivered through the V-IMPACT virtual primary care model was similar to, if not better than, traditional in-person care, especially in terms of medication management and testing compliance.
Robinson et al., 2021 [21]	Secure Messaging in a patient portal	1000	Males: 94 % Females: 6 %	66.4 ± 7.7	51%	Retrospective observational, mixed-methods	Increased secure messaging use was associated with higher odds of achieving HbA1c control (OR = 1.5, 95% CI: 1.2 to 1.8).	Secure messaging can be an effective tool for diabetes self-management, especially in rural populations with limited access to in-person care. This can help in achieving better glycemic control through better communication and support.
Zupa et al., 2022 [13]	Diabetes Care Network (DCN) program for remote diabetes management in rural settings	87	Males: 97.7% Females: 2.3%	67.2 ± 8.35	100%	Prospective cohort study	Significant reduction in HbA1c levels: Erie cohort (-3.03%), Butler cohort (-2.06%).	DCN model effectively improved glycemic control and other clinical outcomes in rural Veterans with type 2 diabetes, demonstrating the feasibility of remote, team-based diabetes specialty care in rural populations.
Zupa et al., 2023 [22]	Telemedicine for endocrinology care in a rural and urban setting	3778	Males: 42% Females: 58%	60.3 ± 12.7	8%	Retrospective cohort study	Telemedicine-only Group: Mean HbA1c Change: -0.06% (no significant change) In-person Care Group: Mean HbA1c Change: -0.37% (95% CI: -0.43 to -0.31, p < 0.001) Mixed Care Group: Mean HbA1c Change: -0.22% (95% CI: -0.28 to -0.16, p < 0.001).	Patients using telemedicine alone had worse glycemic outcomes compared to those using in-person or mixed-care. Among the 297 rural patients, those using telemedicine alone showed no significant HbA1c changes. However, significant HbA1c improvements were seen in rural patients who transitioned to in-person or mixed-care models.
Eiland et al., 2022 [23]	Traditional telehealth visits for endocrinology care in rural settings	139	Females: 57.6% Males: 42.4%	44.5 ± 16.0	100%	Retrospective cohort study	Statistically significant decline in HbA1c, the mean initial HbA1c was 8.4%, and it reduced to 8.0% at the final visit.	Traditional telehealth visits effectively provided long-term care for people with T1D in rural areas, improving glycemic outcomes and demonstrating the potential to remove barriers to care.
Nyenwe et al., 2020 [25]	DC by an endocrinologist vs. DSME by a certified diabetes educator delivered via telemedicine	69	Males: 30% Females: 70%	56.6 ± 8.0	100%	Prospective cohort study	HbA1c reduced from 9.3% to 7.2% in the DC group (p = 0.0002) and from 9.8% to 8.3% in the DSME group (p = 0.009).	Both DC and DSME via telemedicine significantly improved glycemic control in patients with poorly controlled diabetes from rural communities, demonstrating the effectiveness of telemedicine in underserved populations.
Han et al., 2023 [18]	Telemedicine-assisted SMBG vs. traditional BGM in rural areas	418	Males: 45% Females: 55%	52.1 ± 9.2	100%	Open-label randomized clinical trial	HbA1c reduced significantly in the intervention group (7.95% to 7.38%, p < 0.001) but not in the control group (8.03% to 7.98%, p = 0.38).	Telemedicine-assisted structured SMBG significantly improved glycemic control, reduced hypoglycemia risk, and enhanced self-management in rural T2DM patients.
Kobe et al., 2022 [26]	ACDC program, a telehealth intervention for clinic-refractory, uncontrolled T2D in rural areas	230	Males: 95% Females: 5%	59.4 ± 1.1	63%	Mixed-methods implementation study	Mean HbA1c reduced from 9.56% to 8.14% at 6 months, with sustained improvements at 12 and 18 months (p < 0.001).	ACDC significantly improved glycemic control in a previously refractory population, demonstrating the feasibility and effectiveness of comprehensive telehealth interventions in rural areas.

Risk of bias in individual studies

The articles' eligibility was evaluated using various quality appraisal tools. The quality appraisal using the Newcastle-Ottawa scale is shown in Table 3, the Cochrane Risk of Bias in Table 4, and the MMAT in Table 5.

**Table 3:** Quality appraisal using the Newcastle-Ottawa scale (+) indicates a low-quality score in the respective category; (++) indicates a moderate-quality score; (+++) indicates a high-quality score

Study	Selection	Comparability	Outcome
Baum et al., 2023 [19]	+++	++	+++
Davis et al., 2019 [24]	+++	++	++
Lu et al., 2021 [20]	++++	++	+++
Robinson et al., 2021 [21]	++++	++	+++
Zupa et al., 2022 [13]	+++	+	+++
Zupa et al., 2023 [22]	++++	++	+++
Eiland et al., 2022 [23]	+++	++	+++
Nyenwe et al., 2020 [25]	+++	++	+++

**Table 4:** Quality appraisal using the Cochrane Risk of Bias

Study	Random Sequence Generation	Allocation Concealment	Blinding of Participants and Personnel	Blinding of Outcome Assessment	Incomplete Outcome Data	Selective Reporting	Other Bias
Han et al., 2023 [18]	Low risk	Unclear	High risk	Unclear	Low risk	Unclear	Low risk

**Table 5:** Quality appraisal using the Mixed-Methods Appraisal Tool (MMAT)

Study	Is there an adequate rationale for using a mixed-methods design to address the research question?	Are the different components of the study effectively integrated to answer the research question?	Do the different components of the study adhere to the quality criteria of each tradition of the methods involved?	Is the qualitative approach appropriate to answer the research question?	Are the qualitative data collection methods adequate to address the research question?	Are the findings adequately derived from the data?	Are the participants representative of the target population?	Are measurements appropriate regarding both the outcome and intervention (or exposure)?	Are there complete outcome data?
Kobe et al., 2022 [26]	Yes	Yes	Yes	Yes	Yes	Yes	Yes	Yes	Yes

Level of evidence

Although telemedicine has been widely investigated in the past six to seven years [27], our search showed that there is a lack of articles investigating its effectiveness in diabetic patients residing in rural areas. The selected articles in our paper provided a combination of moderate to high evidence. Three articles provided high-certainty evidence, while the remaining seven had moderate evidence.

Baum et al., Zupa et al., and Davis et al. provided a moderate certainty of evidence because their lack of a control group introduces limitations to their conclusion and limits their generalizability [13,19,24]. Although Lu et al. had a large sample size, the male-to-female ratio was predominant (96%), and their observational nature limits its certainty [20]. Robinson et al. conducted a study in which a reliance on participant self-reporting of data with potential bias might arise, such as recall and selection bias decreased their level of evidence [21]. Finally, Eiland et al. and Nyenwe et al. provided moderate certainty of evidence due to their small sample size and selection bias [23,25].

On the other hand, Zupa et al. have strong evidence due to their large sample size and robust statistical methods [22]. Han et al. also provided a strong level of evidence due to their study design being a randomized clinical trial; however, a short follow-up period and selection bias need to be considered [18]. Finally, Kobe et al. have strong evidence for their mixed-method study design, although their single-arm design needs to be considered [26].

Discussion

Summary of Key Findings

Baum et al. conducted a retrospective quality assurance study designed to examine the rapid implementation of telehealth in managing diabetic veterans. Interestingly, 84.2% maintained or improved their HgA1c within one year after the implementation. Moreover, the frequency of patient-provider encounters did not change, indicating that telemedicine will not compromise the patient-provider encounter frequency [19]. Similarly, Kobe et al. used a mixed-method implementation design to assess the effectiveness of the Advanced Comprehensive Diabetes Care (ACDC) program on T2DM. This paper showed significant improvement in glycemic control and, more importantly, sustainability of the outcome. HgA1c levels dropped from a baseline of 9.56 to 8.14 after six months (P-value < 0.001), and this was maintained until 18 months (P-value < 0.001) [26].

Zupa et al., Lu et al., and Eiland et al. focused on telemedicine utilization and patient engagement [20,22,23]. A retrospective cohort study conducted by Zupa et al. compared three patterns of telemedicine intervention. There were three groups: a telemedicine-only group, a usual in-person visit group, and a mixed-method group. Overall, they demonstrated a significant reduction in HbA1c levels for the mixed-care (95% CI, -0.38% to -0.07%; P = .004) and in-person visit groups (95% CI, -0.59% to -0.15%; P < .001) but not for the telemedicine-only group (95% CI, -0.26% to 0.14%; P = .55). Interestingly, patients who exclusively used telemedicine tended to have more comorbidities, be younger, and had the lowest appointment frequency compared to other groups [22]. Lu and colleagues further reinforced these findings with their propensity score-matched cohort study on the effectiveness of the Virtual Integrated Multisite Patient Aligned Care Teams (V-IMPACT) program versus usual care on glycemic control. No significant difference in glycemic control was observed between the V-IMPACT program and traditional usual care. They concluded that overall glycemic control was equivalent and that telemedicine may replace usual care without affecting the quality of care, as indicated by their difference-in-differences estimate of -0.008% (95% CI, -0.055 to 0.039) [20].

Eiland et al. conducted a retrospective cohort study that followed 139 individuals for an average of 32 months. They observed that the mean HbA1c level at baseline was 8.4%, significantly decreasing to 8% (P < 0.001). Not only that, but patients with poorly controlled T1D (HbA1c > 9%) had a more significant decline. The magnitude of this fall in HbA1c levels was even observed among patients who used to be seen by an endocrinologist, which may be attributed to the more frequent patient-provider encounters [23]. Another study by Nyenwe et al. compared two intervention groups that received either diabetes consultation (DC) or diabetes self-care management education (DSME) through telemedicine. The two intervention groups recorded significant reductions in HbA1c levels, with the DC group reduced from 9.3 ± 1.3% to 7.2 ± 0.9% (P = 0.0002) and DSME reduced from 9.8 ± 1.6% to 8.3 ± 1.9% (P = 0.009) [25].

The significance of the structured and collaborative approach has been emphasized in three articles: Zupa et al., Han et al., and Robinson et al. [13,18,21]. Zupa et al. evaluated an already-established innovative intervention in a veterans' health system, the Diabetes Care Network (DCN), which proposes remote self-management education for veterans with T2DM living in rural settings. This study observed significant reductions in HbA1c levels, with decreases of 3.03% (P < 0.001) in the Erie cohort and 2.06% (P < 0.001) in the Butler cohort over 12 months [13]. Similarly, Han and colleagues conducted a single-blinded, randomized trial with a 1:1 increment to evaluate whether telemedicine-assisted structured self-monitoring of blood glucose (SMBG) is more effective than standard blood glucose meter (BGM). The intervention group recorded a significant decrease in HbA1c levels from 7.95% to 7.38% (P < 0.001). Additionally, the studies reported a reduced risk of hypoglycemia and improved diabetes self-management behaviors [18]. Further, Robinson et al. supported these findings by revealing that secure messaging (SM) significantly improved diabetes self-management in rural settings, and thus, significant glycemic control was achieved [21]. Finally, the Mississippi Diabetes Telehealth Network study by Davis et al. illustrates the impact of remote patient monitoring (RPM) and telehealth on diabetes management in rural areas. The study observed an improvement in HbA1c levels, beginning with a baseline of 9.5%, which steadily decreased to 7.9% over 12 months (P < 0.001) [24].

Comparative Analysis of the Results Across the Studies

A significant reduction in HbA1c levels was observed in most studies, indicating better glycemic control and patient engagement. For example, Kobe et al. reported a significant decrease in HbA1c levels, from 9.56% to 8.14% (−1.43%, 95% CI: −1.64 to −1.21; P < .001) over six months, and these improvements were sustained over 18 months (−1.08%, 95% CI: −1.35 to −0.81; P < .001). This study involved 230 patients across seven Veterans Health Administration sites, utilizing telemonitoring in self-management support [26]. The number of telehealth visits was about the same as the previous face-to-face encounter, suggesting strong patient engagement. This indicates that telemedicine can effectively replace or complement traditional in-person visits.

Zupa et al. introduced the DCN, a comprehensive health initiative that delivered diabetes care through home telehealth monitoring. Local rural liaisons provided personalized feedback on glucose levels to patients with T2DM. Over 12 months, the Erie cohort saw a 3.03% reduction in HbA1c levels and a 2.06% decrease in the Butler cohort [13]. Similarly, Han et al. implemented a telemedicine program that enabled patients to manage their blood sugar levels using an app for daily real-time tracking and weekly tele-coaching. This intervention resulted in a significant HbA1c reduction from 7.95% to 7.38% (P < 0.001) over six months, compared to a nonsignificant decrease from 8.03% to 7.98% (P = 0.38) in the control group [18]. Both of these papers show the effectiveness of telemedicine when integrated into a structured program.

However, some outcome variation might arise even when telemedicine is integrated into a structured program. For example, Lu et al. did not find significant differences in HbA1c levels between the V-IMPACT telehealth program and usual care. Yet, there were improvements in medication adherence and quality indicators. The study involved 9,010 veterans and aimed to integrate telehealth into existing healthcare systems, focusing on adherence to diabetes quality indicators. The study design included propensity score matching to compare outcomes between the telehealth and usual care groups. There was no statistically significant decrease in HbA1c levels in the intervention group [20]. This outcome might be attributed to several factors: both groups were well-matched at baseline, ensuring no significant demographic or clinical differences influenced the results; the usual care provided by the Department of Veterans Affairs was already of high quality, effectively managing diabetes in both groups; both groups maintained high engagement with primary care services, including frequent visits and routine monitoring; and both models of care included comprehensive diabetes management, such as medication monitoring and patient education. These factors suggest that while telehealth can improve certain aspects of diabetes management, its impact on glycemic control may vary depending on the specific implementation and patient engagement levels. Because all patients involved in this study were already in a high-quality healthcare system, the reason for similar findings is the well-established system rather than the mode of communication.

Sustained patient-provider encounters and local support systems are essential for successful telehealth implementation. Eiland et al. demonstrated this in their retrospective cohort study by tracking type 1 diabetic patients for 32 weeks. They had regular telehealth check-ins, continuous glucose monitoring, and real-time treatment adjustments. By the end of the study, the patients' average HbA1c levels significantly dropped from 8.4% to 8% (P < .001) [23]. In Nyenwe et al.'s study, the participants in five rural communities in Tennessee were split into two groups: one received DC and the other DSME. They connected with an endocrinologist via videoconference for medical history reviews, visual clinical evaluations, and basic physical exams conducted by a nurse under the endocrinologist's guidance. After a year, both groups experienced significant drops in HbA1c levels, showing that a comprehensive approach supported by the local community can effectively help manage and improve glycemic control in rural areas [25]. Both studies demonstrate that frequent and consistent interaction can lead to better glycemic control.

Telemedicine can be used in different ways to achieve better glycemic control, and one of the ways that has been investigated is to increase patient awareness. Robinson et al. stated that diabetic patients in rural areas are more likely to use telehealth services than those in urban areas. They conducted a retrospective observational cohort study with mixed methods to assess if SM could help improve glycemic control through better diabetes self-management. They concluded in the study that more frequent SM use was associated with better diabetes self-management (P < 0.007) and, as a result, better glycemic control (P < 0.001). This indirect effect was seen in diabetic patients living in rural areas (95% CI 0.004-0.927) but not in urban areas (95% CI -1.039 to 0.056). Moreover, rural patients frequently used this method to discuss and share their diabetes health issues (77% vs. 67% P = 0.01), suggesting that these interventions are viewed positively in underserved areas [21].

Additionally, Davis et al. assessed the efficiency of the Mississippi Diabetes Telehealth Network in enhancing diabetes self-management among rural populations by conducting educational sessions as they conducted a prospective, longitudinal cohort design involving 115 participants. Three months after the implementation, the HgA1c levels dropped from a mean of 9.5% to 7.7%, and most importantly, this was sustained until the last follow-up at 12 months, where the mean HgA1c level was 7.7% (P < 0.001) [24]. These studies emphasized the importance of patient empowerment in diabetes management, demonstrating that telemedicine can effectively enhance glycemic control by encouraging patients to take an active role in their care.

Despite the previous positive results and findings supporting telemedicine, some studies reported challenges and limitations in implementing this intervention. For example, Baum et al. found that only 12.3% had a decreased HbA1c level, whereas 15.7% had worsened control, and 72% maintained their HbA1c level [19]. The reasons for these outcomes might be the rapid implementation with no structured approach, limited access to nutritious food, and the psychological impact of the pandemic. Similarly, Zupa et al. conducted a retrospective cohort study to compare the effectiveness of telemedicine, in-person, and mixed-care modalities in achieving better glycemic control. The study found that patients using telemedicine alone had no significant change in HbA1c at 12 months (-0.06%; 95% CI, -0.26% to 0.14%; P = .55). In contrast, the in-person cohort showed a significant HbA1c reduction of -0.37% (95% CI, -0.59% to -0.15%; P < .001), and the mixed cohort showed a reduction of -0.22% (95% CI, -0.38% to -0.07%; P = .004) [22]. Both studies indicate that while telemedicine can bridge the gap between patients and clinicians, its effect on glycemic control remains inconclusive, particularly for patients who rely solely on telemedicine.

Implications for Clinical Practice of Telemedicine in Rural Areas

Telemedicine is becoming a crucial innovation, particularly in rural and remote areas, for managing various chronic diseases, including diabetes [28]. However, the clinical trials and studies conducted in rural areas are fewer than those conducted in urban areas [29]. Thus, the clinical implications need to be considered cautiously. Our analysis revealed variation in the results depending on the study designs, and there are no established criteria to definitively evaluate telemedicine's success.

Telemedicine has shown promising results and can achieve better outcomes when integrated into structured and collaborative programs. Zupa et al. illustrated this by integrating telemedicine diabetic care with local primary liaisons. Their approach, which incorporated real-time data input and collaborative care from nurses and HCPs, significantly decreased HbA1c levels in the intervention groups [13]. However, Lu et al. discovered no significant difference in HbA1c levels between the control and intervention groups. It is worth mentioning that both groups had decreased levels of HbA1c, but the difference between them was insignificant. Nevertheless, their research demonstrated that the V-IMPACT program has the potential to enhance the overall care of diabetes patients in terms of medication adherence and regular testing. The intervention group had significant differences in adherence to medications such as statins, ACE/ARBs, and annual microalbumin testing compared to the typical in-person visit [20]. These data indicate that although there may not be a difference in glycemic control, telemedicine offers advantages regarding medication adherence and compliance with quality indicators, ultimately resulting in improved health outcomes.

The implication of such an implementation needs to be seen holistically; one of the most challenging aspects of rural health is that each rural environment has its unique demographics, culture, and challenges [30]. According to Davis et al., numerous rural regions require more infrastructure to support establishing high-quality telehealth services. Moreover, diabetic patients with complex comorbidities may not benefit as much from telemedicine only as they require conventional in-person care [24]. Zupa et al. observed that the group exclusively using telemedicine did not significantly reduce HbA1c levels compared to the other two groups. The less significant decrease in HbA1c in the telemedicine-only group resulted from the complex comorbidities [22]. Han et al. also supported this by observing that patients with multiple comorbidities require a hybrid approach that combines telehealth with traditional in-person visits [18]. Furthermore, in Zupa et al.'s study, it is essential to consider the frequency of appointments, as the group that only used telemedicine in this study had fewer appointments than the other two groups [22]. These studies' results suggest that telemedicine success metrics should not be limited to HbA1c levels alone. The findings from these studies indicate that the success metrics for telemedicine should extend beyond just HbA1c levels. Metrics such as patient satisfaction, quality of life, and overall health outcomes are equally important in evaluating its effectiveness.

Another challenge that might interfere with the feasibility of telemedicine implementation is the increased workload on HCPs, which could explain the lower appointment frequency in the telemedicine-only group in the Zupa et al. study [22]. This issue was noted by Baum et al. and Kobe et al.; even though telemedicine showed maintenance or improved glycemic control, the higher workload on HCPs could affect its feasibility for long-term use [19,26]. Further studies are needed to implement strategies to streamline telehealth processes, avoid HCP burnout, ensure long-term sustainability, and find ways to integrate telemedicine into existing workflows seamlessly.

Limitations

In this systematic review, we faced challenges in finding articles that assess the efficiency of telemedicine in rural areas due to the lack of studies on this topic in the literature. Among those included, there was diverse heterogeneity in study design, intervention types, and participant characteristics, making comparisons between the studies more challenging. For instance, while some studies, such as Eiland et al., Han et al., and Robinson et al., reported significant reductions in HbA1c levels, others, such as Lu et al. and Baum et al., did not [18-21,23]. These discrepancies between the results may be due to variations in the intensity of the intervention, sample demographics, and different telemedicine approaches.

A second consideration is the small sample sizes in several studies, which limit the broader application of the findings. For instance, Kobe et al., Zupa et al., Nyenwe et al., and Eiland et al. conducted studies with a limited number of participants that might not accurately represent the wider diabetes patient population in rural areas [13,23,25,26]. The follow-up durations differed significantly between studies, ranging from a few months to 32 months [18,23]. Shorter monitoring periods might not be sufficient to assess telemedicine’s long-term feasibility and sustainability.

Finally, many studies still need to address the unique challenges of the rural areas where the interventions were conducted. Infrastructure and the distinct cultural environments of each rural area must be explored, as they might influence people's willingness to use telemedicine for diabetes self-management. Addressing these limitations in future research is crucial. It will require larger sample sizes, standardized outcome measures, and a focus on the specific needs and conditions of rural populations to better understand the full potential and limitations of telemedicine.

## Conclusions

In conclusion, this systematic review demonstrates that telemedicine significantly enhances glycemic control for diabetic patients in rural areas by improving HbA1c levels, enhancing medication adherence, and providing timely support. The success of telemedicine relies on structured programs tailored to local challenges, considering infrastructure, cultural variations, and HCPs' workloads. For patients with complex comorbidities, integrating traditional visits with telemedicine is essential. Innovative solutions, such as offline data collection and low-bandwidth technologies, need further investigation. Additionally, more studies are required to assess broader success metrics, such as quality of life and patient satisfaction. Future research should focus on large-scale randomized trials to evaluate and validate telemedicine's effectiveness and sustainability.
